# Population Genomics of Secondary Contact

**DOI:** 10.3390/genes1010124

**Published:** 2010-06-25

**Authors:** Anthony Geneva, Daniel Garrigan

**Affiliations:** Department of Biology, University of Rochester, Rochester, New York, USA; E-Mail: anthony.geneva@rochester.edu

**Keywords:** coalescent, haplotype, hybridization, introgression

## Abstract

One common form of reticulate evolution arises as a consequence of secondary contact between previously allopatric populations. Using extensive coalescent simulations, we describe the conditions for, and extent of, the introgression of genetic material into the genome of a colonizing population from an endemic population. The simulated coalescent histories are sampled from models that describe the evolution of entire chromosomes, thereby allowing the expected length of introgressed haplotypes to be estimated. The results indicate that our ability to identify reticulate evolution from genetic data is highly variable and depends critically upon the duration of the period of allopatry, the timing of the secondary contact event, as well as the sizes of the populations at the time of contact. One particularly interesting result arises when secondary contact occurs close to the time of a severe founder event, in this case, genetic introgression can be substantially more difficult to detect. However, if secondary contact occurs after such a founding event, when the range of the colonizing population increases, introgression is more readily detectable across the genome. This result may have important implications for our ability to detect introgression between ancestrally bottlenecked modern human populations and archaic hominin species, such as Neanderthals.

## 1. Introduction

The proposition that a "web of life" is a more appropriate metaphor for the evolution of biodiversity than a strictly bifurcating "tree of life" is supported by a growing body of evidence for genetic exchange between populations from a wide range of taxonomic groups [1]. A subset of this empirical evidence also supports an important role for reticulate evolution in the processes of adaptation and speciation [2]. Introgressed genomic regions that confer a selective advantage to the host genome are expected to spread rapidly from one population to the next. Alternatively, genomic regions involved in reproductive isolation will be severely hindered in their ability to move between populations. To construct robust statistical tests of hypotheses that posit roles for natural selection and reproductive isolation during genetic exchange between populations, it is first necessary to understand fully how introgression occurs in the absence of these forces. It is first important to recognize that reticulate evolutionary patterns may result from a variety of different population-level processes. For example, genetic introgression may occur due to the presence of either a stable or transitory hybrid zone between populations [3,4,5]. However, another potentially widespread process leading to reticulate evolutionary patterns may be that of discrete secondary contact events between previously allopatric populations. This form of reticulate evolution may be important for continental or oceanic populations with long-range dispersal [6], island or freshwater populations [7], and terrestrial populations relegated to glacial refugia during the Pleistocene [8].

The basis for inferring genetic introgression in phylogeographic studies has been largely limited to the hierarchical clustering of gene tree lineages, which are reconstructed from samples drawn from two or more populations of interest [9,10]. However, such inferences can be easily confounded and tend to suffer from an overall lack of statistical power [11,12]. A more detailed treatment of the problem can be afforded by incorporating population genetics theory. Several population genetics tools, designed to detect introgression or gene flow between populations, already exist and enjoy widespread usage [13,14]. Despite such methodological advances, one important area of investigation focuses on the power of such methods to detect genetic introgression. For example, if one assumes that hybridization has occurred, how likely are we to detect it with a particular sample? Or, how readily can a locus reflecting the hybridization event be randomly sampled from the genome? The answer to these questions depends upon the timing of the putative hybridization event(s), the amount of divergence between the populations, the rate of hybridization, and the effective sizes of the populations. Can population genetics tools be leveraged to obtain estimates of these parameters? If so, the study of genetic introgression may progress from qualitatively determining whether or not it has occurred to asking more specific questions, such as is hybridization more likely to occur early in sympatry, when the density of an invading population is low, or later as its density and range increases?

Studying genetic introgression that arises as a consequence of secondary contact is of particular interest because it is likely to be a major source of reticulate evolution. However, secondary contact may also generate the most readily identifiable pattern of reticulate evolution using DNA sequence data and can be detected with a sample drawn from a single population [15,16]. This latter advantage may be important for identifying situations in which extinction via hybridization has occurred [17].

**Figure 1:**
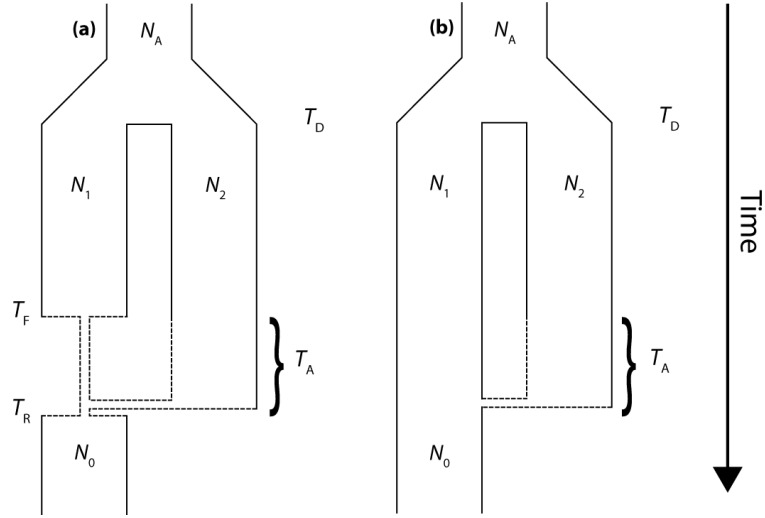
(a) The Isolation-and-Admixture (IAA) model with a founder event, (b) the IAA model with constant population size. See Table 1 for a detailed explanation of model parameters.

The present work utilizes coalescent-based modeling techniques to characterize the properties of gene trees that result from secondary contact. We employ a simple "Isolation-and-Admixture" (IAA) model [18] to generate a series of partially linked gene trees along chromosomes to determine the expected length of introgressed haplotypes. The IAA model describes the ancestry of a sample of chromosomes taken from the colonizing population and includes a period of admixture with an endemic population, from which it diverged from an ancestral population at some point in the past. The present implementation of the IAA model also includes a scenario in which the colonizing population undergoes a founder event (Figure 1). The general behavior of the IAA model is governed by ten demographic parameters that are explained in Table 1.

Two summary statistics, describing the total depth of gene trees (*T*_MRCA_) and the proportion of the total tree length that can be mapped to the two basal branches (*P*_BB_), are measured under different parameterizations of the IAA model to identify evolutionary scenarios in which introgression can be most readily detected. The average level of autocorrelation of these two statistics is measured along chromosomes, as a proxy for linkage disequilibrium [19]. The effective number of independent gene trees along a chromosome is proportional to the probability that an investigator will sample a region containing an introgressed haplotype. This genomic sampling distribution for introgressed haplotypes will become increasingly important as investigators begin to accumulate next-generation resequencing datasets. One general finding to come out of this work is that introgression is very difficult to detect if hybridization occurs close to the time of the founding of one of the populations.

**Table 1:** Description of the parameters of the ten parameters of the Isolation-and-Admixture (IAA) model and the two variables used to measure the properties of genetic variation expected under the model.

Parameter/Variable	Description
*T*_D_	Original divergence time between the two species or populations
*T*_F_	Time of the founder event for the colonizing species/population
*T*_R_	Time of recovery for the effective size of the colonizing population
*T*_A_	Time of the admixture event
*N*_A_	Effective size of the ancestral population
*N*_1_	Effective size of the source population for the colonizing population
*N*_2_	Effective size of the endemic population
*N*_0_	Current effective size of the colonizing population
*c*	Admixture proportion between the colonizing and endemic populations
α	The effective size of the founding population relative to that of the source population
*P*_BB_	The proportion of total gene tree branch length occupied by the two basal branches
*T*_MRCA_	The time to a most recent common ancestor for a sample from the colonizing population
*L*[*x*]	The autocorrelation coefficient between variables at distance, or lag, *x*

## 2. Results and Discussion

The coalescent simulations of the IAA model are designed to determine systematically how parameters such as population divergence time, the timing and rate of admixture, and the magnitude of a founder event influence the structure and depth of gene trees that can be sampled from a genome. We do not explicitly model mutational processes and, therefore, do not deal directly with haplotype sequence data. Instead, we characterize two specific properties of gene trees that have important consequences for the patterns haplotype sequence diversity that may be sampled from a single population. Often investigators who wish to address questions of hybridization and gene flow will obtain sequence data from two or more populations to look for shared mutations or reciprocal monophyly in the resulting gene trees. However, we consider only the ability to detect hybridization and introgression using a single population sample. The rationale for this approach is that it does not rely on specific assumptions about which populations may actually be exchanging genes, or even whether a second population still exists.

**Figure 2:**
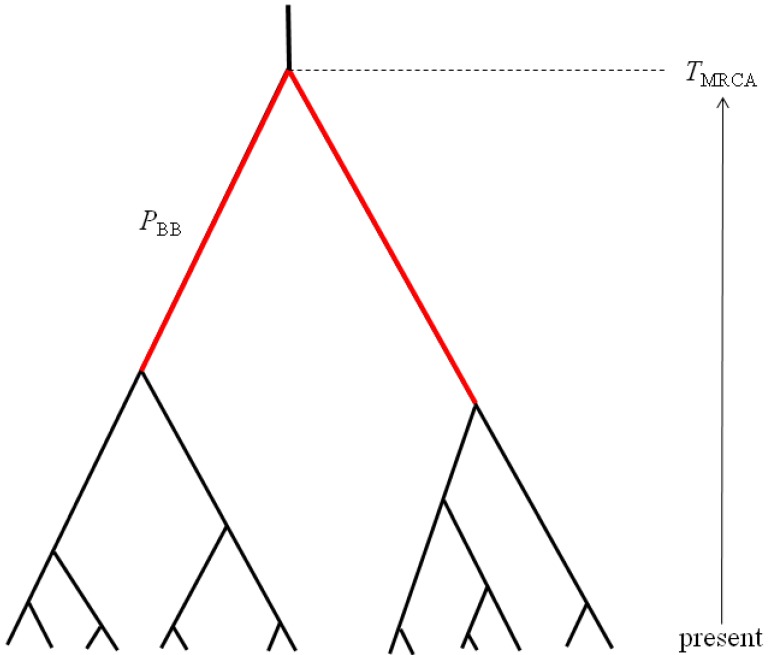
The two summaries of gene tree shape measured from simulations of the IAA model. *T*_MRCA_ is the total height of the gene tree and is expected to be deeper when admixture occurs. *P*_BB_ is the proportion of the total tree length that is occupied by the two basal branches shown in red.

The first property of sampled gene trees that we consider is summarized by the statistic *P*_BB_, which can be defined as the proportion of the total gene tree branch length that is occupied by the two basal branches (Figure 2). Mutations occurring along these two basal branches serve as the basis for a statistical test for detecting hybridization from single population samples [16]. The second gene tree property of interest is the *T*_MRCA_, which is proportional to maximum number of sequence differences among a sample of haplotypes (Figure 2). These two properties are chosen to be complementary summaries of the gene trees that are expected to arise under the IAA model. We examine the statistical behavior of these two properties under a range of parameterizations of the IAA model by varying divergence time, admixture time, the magnitude of a putative founder event, and the rate at which admixture occurs. Lastly, entire chromosomes are simulated to estimate the length of introgressed genomic regions. Although the sampled model parameters and summary statistics are necessarily limited in scope, the results provide significant insight into the behavior of models of secondary contact and what patterns investigators might expect to find in their sequence diversity datasets.

### 2.1. Isolation and Admixture Model

The molecular signature of genetic introgression due to secondary contact that we consider here is a partitioning of the ancestral lineages of a single population gene tree into two highly divergent clusters. Looking backwards in time, this corresponds to the free coalescence of genes sampled from a population, until the time of admixture, *T*_A_. Then at time *T*_A_, the remaining ancestral lineages are partitioned into two ancestral populations and the two clusters of lineages are no longer exchangeable, until the original population divergence time *T*_D_ (furthermore, it is assumed that *T*_D_ >> *T*_A_). The loss of exchangeability and the difference between *T*_D_ and *T*_A_ is expected to result in elongated basal branches for resulting gene trees. If no admixture occurs, the gene tree will conform to the expectations from a single, panmictic population, and *T*_D_ and *T*_A_ will have no effect on the gene tree. If admixture occurs (and, hence, imposes the loss of exchangeability during the ancestral process), both the relative length of the two basal branches (*P*_BB_) and the depth of the gene tree (*T*_MRCA_) are expected to increase, compared to a model for which the rate of admixture is zero.

**Figure 3:**
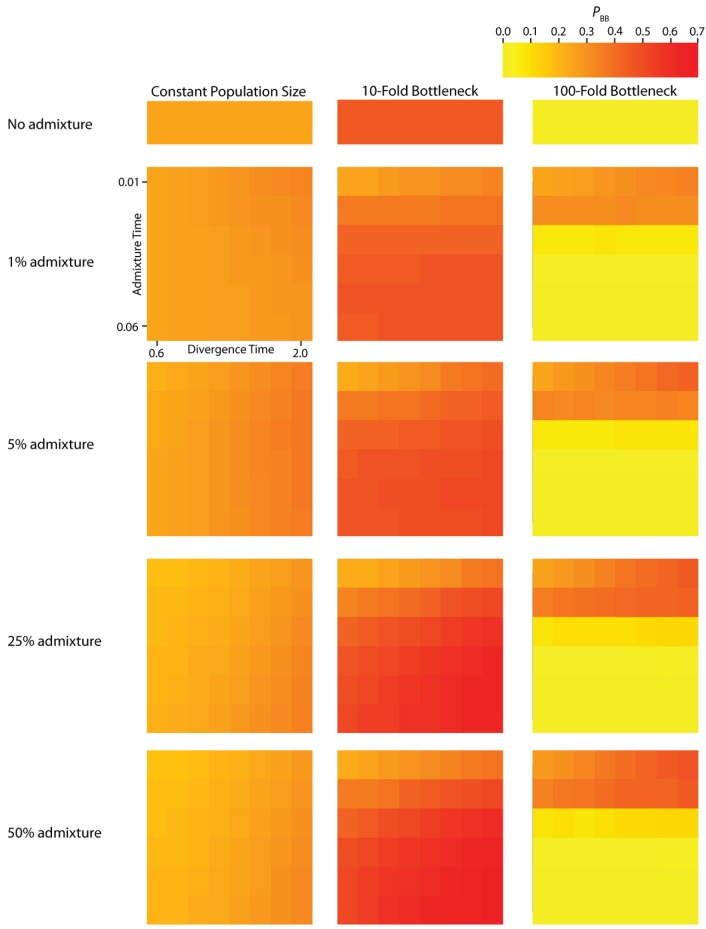
Simulated values for the proportion of total gene tree length that occurs in the two basal branches, or *P*_BB_. Each column corresponds to a different magnitude of founder event in the colonizing population. Each row represents differing levels of admixture between the colonizing and endemic populations. Finally, within each panel, squares correspond to different combinations of population divergence time and admixture time in the IAA model.

**Figure 4:**
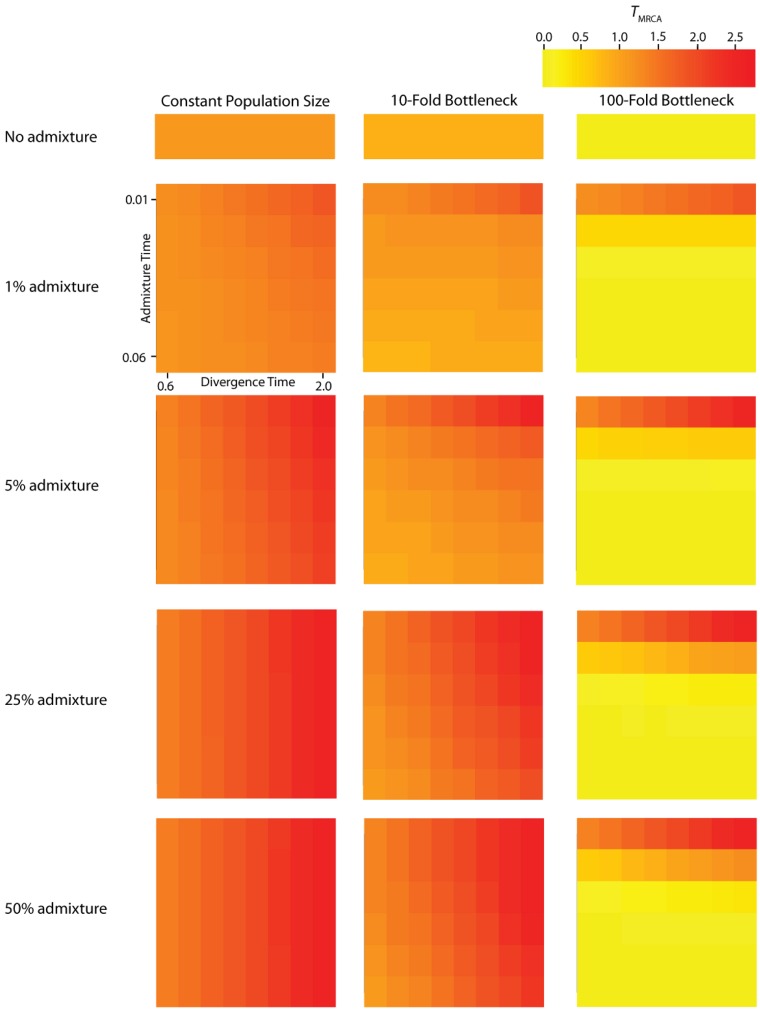
Simulated values for the height of genes trees, or *T*_MRCA_. Each column corresponds to a different magnitude of founder event in the colonizing population. Each row represents differing levels of admixture between the colonizing and endemic populations. Finally, within each panel, squares correspond to different combinations of population divergence time and admixture time in the IAA model.

To assess the relative contribution of each IAA parameter to variation in *P*_BB_ and *T*_MRCA_, we perform a one-way analyses of variance (ANOVA, Table 2). Furthermore, to assess how parameters of the IAA model affect the distribution of gene trees across the genome, the effective number of independent gene trees (*G*_e_) is also calculated (Table 3). The *r^2^* values resulting from these analyses determine the proportion of total variation in a summary statistic that can be explained by varying each parameter individually, as well as by the interaction of parameters. Lastly, the residuals for each analysis reflect the stochastic nature of the underlying coalescence process. The average *T*_MRCA_ (Figure 3) and *P*_BB_ (Figure 4) vary substantially across all of the simulated parameterizations of the IAA model. Most notably, *T*_MRCA_ and *P*_BB_ are primarily influenced by the time of divergence (*T*_D_). This result is not necessarily surprising since basal branches are longest when the time of isolation (the difference in *T*_D_ and *T*_A_) is greatest. In our simulations, this difference is primarily determined by the value of *T*_D_. The other major contributor to variation in *T*_MRCA_ and *P*_BB_ is the proportion of admixture (*c*).

**Table 2:** Analysis of variance for IAA model parameters for constant size population (α = 1), mild founder event (α = 0.1), and strong founder event (α = 0.01). See **Table 1** for detailed explanation of parameters).

	α = 1		α = 0.1		α = 0.01	
Parameter	*P*_BB_	*T*_MRCA_	*P*_BB_	*T*_MRCA_	*P*_BB_	*T*_MRCA_
*T*_A_	0.0221	0.0114	0.5734	0.1679	0.7425	0.6332
*T*_D_	0.6154	0.5556	0.1142	0.2828	0.0074	0.0148
*c*	0.2026	0.2077	0.1137	0.3492	0.0065	0.0073
*T*_A_ × *T*_D_	0.0008	0.0020	0.0041	0.0098	0.0110	0.0227
*T*_A_ × *c*	0.0233	0.0081	0.0306	0.0100	0.0060	0.0068
*T*_D_ × *c*	0.0103	0.0341	0.0262	0.0683	0.0010	0.0018
*T*_A_ × *T*_D_ × *c*	0.0039	0.0018	0.0012	0.0019	0.0008	0.0015
Residuals *^a^*	0.1216	0.1793	0.1367	0.1101	0.2249	0.3118

*^a^* Residuals reflect stochastic variation inherent in the coalescent process.

We consider three different implementations of the IAA model: 1) constant size of the colonizing population, 2) a 10-fold founder event, and 3) a 100-fold founder event in the colonizing population. In the case of constant population size, the *T*_D_ parameter exerts the greatest influence on gene trees, as summarized by both *P*_BB_ and *T*_MRCA_. For example, *T*_D_ explains ~62% of the variation in *P*_BB_ and ~56% of the variation in *T*_MRCA_. This result is expected because the proportion of the gene tree occupied by the basal branches is primarily constrained by the time of divergence. Similarly, the depth of a gene tree is directly influenced by the *T*_D_ parameter, which governs the exchangeability of lineages. Admixture proportion also exerts a strong influence on the sampling distribution of gene trees. The admixture parameter, *c*, explains ~20% of the variation in *P*_BB_ and ~21% of the variation in *T*_MRCA_. The admixture rate is influential because it governs the partitioning of the ancestral lineages into the two ancestral populations. A larger admixture rate will more evenly allocate the lineages and, thus, decrease *P*_BB_. This same allocation process causes more ancestral lineages to survive until time *T*_D_, thereby having the effect of increasing the T_MRCA_. Finally, ~12% of the variation in *P*_BB_ and ~18% of the variation in T_MRCA_ is attributable to stochasticity inherent in the coalescent process.

**Table 3:** Analysis of variance for the effective number of gene trees (*G*_e_) for constant size population (α = 1), mild founder event (α = 0.1), and strong founder event (α = 0.01). See Table 1 for a detailed description of the IAA model parameters.

Parameter	α = 1	α = 0.1	α = 0.01
*T*_A_	0.0181	0.8889	0.4604
*T*_D_	0.3975	0.0351	0.0013
*c*	0.4708	0.0129	0.0000
*T*_A_ × *T*_D_	0.0000	0.0220	0.0027
*T*_A_ × *c*	0.0019	0.0006	0.0001
*T*_D_ × *c*	0.0000	0.0025	0.0000
*T*_A_ × *T*_D_ × *c*	0.0016	0.0005	0.0001
Residuals *^a^*	0.1100	0.0375	0.5354

*^a^* Residuals reflect stochastic variation inherent in the coalescent process.

### 2.2. The Effect of a Founder Event

Across all simulations, the parameter that has the largest influence on both *P*_BB_ and *T*_MRCA_ is the magnitude of the founder event (α). When the founder event is extreme (α = 0.01), both the mean and variance of *P*_BB_ and *T*_MRCA_ are substantially decreased. A strong founder event reduces the variance of the genomic distribution of gene trees and leads to an increased level of spatial autocorrelation at lag 25 trees (*L* [25]). Interestingly, a milder founder event has the opposite effect, whereupon both the mean and variance of *P*_BB_ and *T*_MRCA_ are increased relative to the constant population size case. Likewise, for both measures, *L* [25] is decreased relative to a constant population size, indicating a higher variance in these values across a chromosome. One exception to this pattern occurs when admixture is very recent (*T*_A_ is close to *T*_R_), in which case the ancestral process converges to the constant population size case. *P*_BB_ is maximized under demographic scenarios for which each population coalesces to a single ancestor during, or shortly before, the founder event (*e.g*., the earliest possible time when one lineage remains in each of the two populations). Under an extreme founder event, often all coalescent events occur prior to admixture (resulting in an exceedingly recent *T*_MRCA_). In these cases, no introgressed gene regions would be detectable in the sample, despite the occurrence of historical admixture. Under a mild founder event, the opposite appears true: a smaller number of ancestral lineages remaining at time *T*_A_ are partitioned into the two populations, thereby maximizing *P*_BB_ and *T*_MRCA_.

The introduction of a mild founder event also substantially alters the relative effects of other demographic parameters. When α = 0.1, variation in *T*_A_ has the greatest influence on gene trees, accounting for ~57% of the variation in *P*_BB_ and ~17% of the variation in *T*_MRCA_. The actual rate of admixture accounts for ~11% of the variation in *P*_BB_ and ~35% of the variation in *T*_MRCA_. The interaction of these two parameters lies at the heart of detecting introgression. Because the power to detect admixture using genetic data relies upon ancestral lineages descending from at least two historically allopatric populations, the two most important factors are (1) how many ancestral lineages remain at the time of the admixture events and (2) the probability that a lineage is moved to the other population. A strong founder event usually results in only a single ancestral lineage persisting until the time of the admixture event. In this case, it does not matter if that single ancestral lineage changes resident populations, it will have no effect on the gene tree. However, the most reliable way to generate a signal of introgression is when only two ancestral lineages remain at the time of the admixture event and one changes residence, while the other does not. In this case, *P*_BB_ is maximized, and this scenario is most easily generated under a mild founder event implementation of the IAA model.

### 2.3. The Size of Introgressed Haplotypes

**Figure 5:**
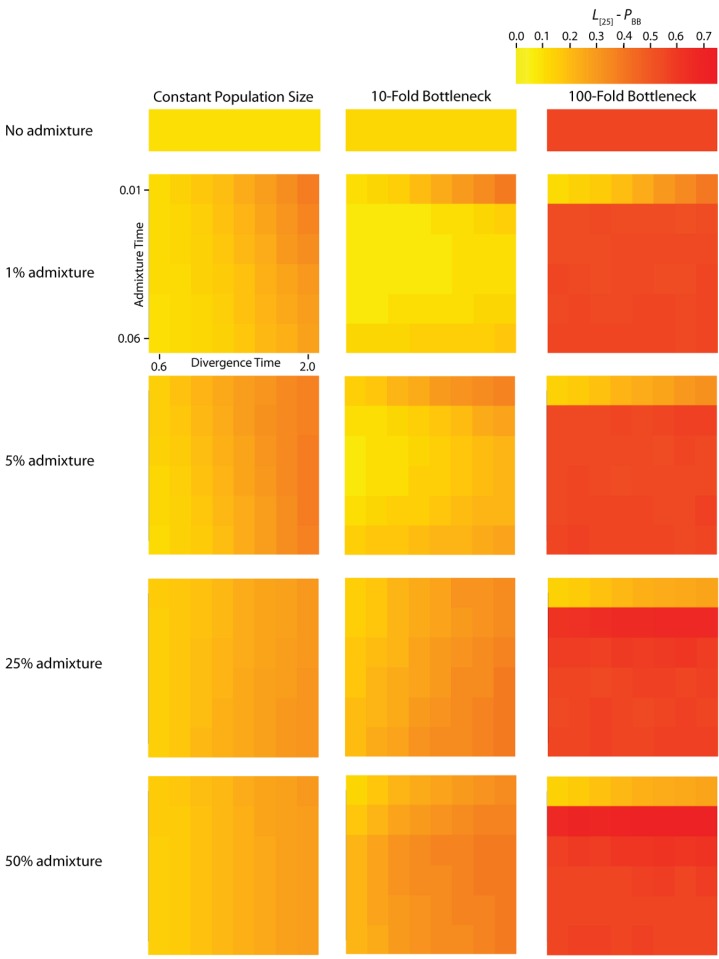
Simulated autocorrelation coefficients for *P*_BB_ at a lag of 25 gene trees. Each column corresponds to a different magnitude of founder event in the colonizing population. Each row represents differing levels of admixture between the colonizing and endemic populations. Finally, within each panel, squares correspond to different combinations of population divergence time and admixture time in the IAA model.

**Figure 6:**
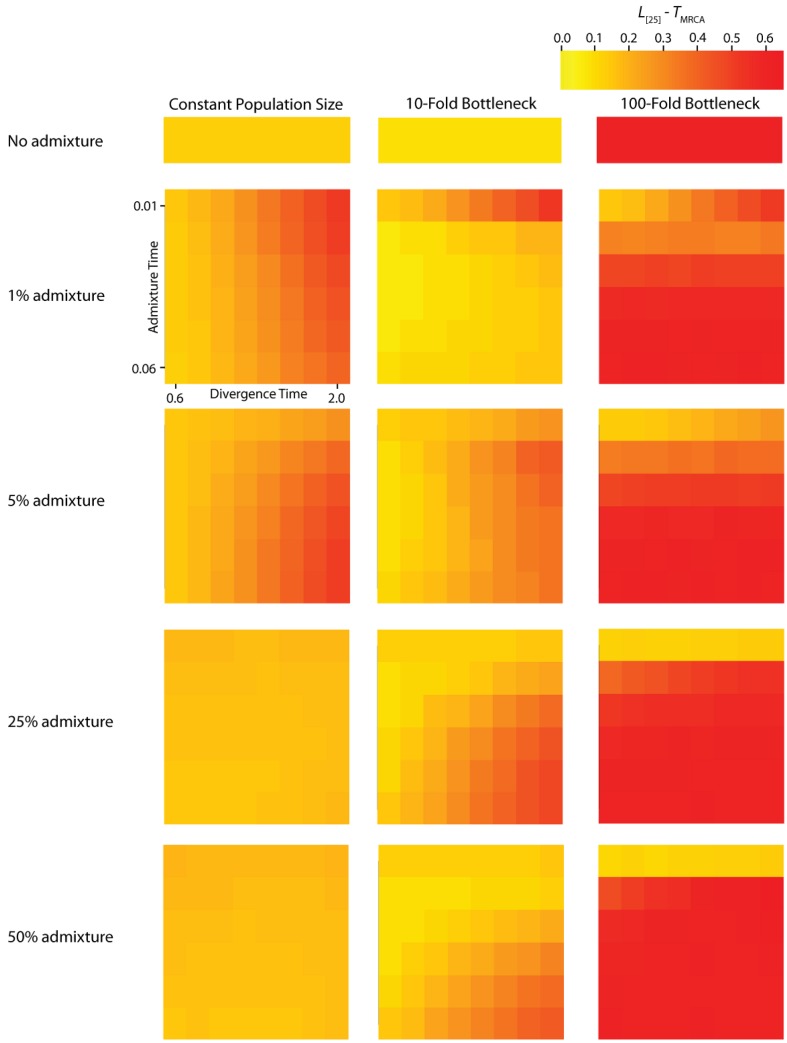
Simulated autocorrelation coefficients for *T*_MRCA_ at a lag of 25 gene trees. Each column corresponds to a different magnitude of founder event in the colonizing population. Each row represents differing levels of admixture between the colonizing and endemic populations. Finally, within each panel, squares correspond to different combinations of population divergence time and admixture time in the IAA model.

A single simulated dataset comprises a variable number of gene trees (*G*). The number of gene trees is exponentially distributed with mean *nρ*/2, where *n* is the number of sampled chromosomes and *ρ* = 4*Nr* and *r* is the rate of crossing-over per generation between the ends of the simulated chromosome. The expected number of gene trees for a particular IAA model parameterization is measured by calculating the mean number of gene trees generated per replicate across all replicates. However, adjacent gene trees, which differ by a single recombination event, can have highly correlated topologies [20] and therefore may not differ with respect to *P*_BB_ and *T*_MRCA_. It is noteworthy that any given recombination event has the potential to either eliminate an introgression event from the previous gene tree or add an introgression event to a gene tree that previously did not have any. The average autocorrelation coefficient for both *P*_BB_ and *T*_MRCA_ at varying lag values contains information about the average length of introgressed haplotypes. The calculation of *L* [25] autocorrelation of *P*_BB_ (Figure 5) and *T*_MRCA_ (Figure 6) provides a measure of the relationship of gene trees across a chromosome. However, the level of autocorrelation can also be influenced by a reduction in the variance of the summary statistics. When values of *P*_BB_ or *T*_MRCA_ are constrained due to demographic parameters, *L* [25] increases.

The effective number of gene trees (*G*_e_) is the number of effectively independent gene trees that can be sampled from a chromosome and this quantity can be directly estimated from the first-order autocorrelation coefficient (eq. (4)). It should also be noted that we do not explicitly consider the physical distance between gene trees arrayed along chromosomes, but instead only the linear arrangement of gene trees along chromosomes. The ANOVA results for these data (Table 2) indicate that most of the variation in *G*_e_ is due to variation in the population size during a founder event. In all of the simulations, recombination was modeled along a hypothetical chromosome (or chromosome segment) containing 10^6^ loci. The actual number of gene trees reported, *G*, is substantially less than 10^6^, because adjacent loci that do not experience a recombination event between them have identical gene trees (data not shown). Consequently, variation in the total number of gene trees between constant and founder event populations is due to the effect varying α has on the effective population size over time and total coalescent time in the simulations.

For the IAA simulations that model a constant effective population size, *G*_e_ is primarily influenced by the *c* parameter (~47% of the variation). In a constant-sized population, moderate to high levels of admixture (*c* = {0.25, 0.5}) decreases the autocorrelation between adjacent gene trees (Figure 5) and consequently increases *G*_e_. The time of divergence (*T*_D_) also affects the autocorrelation— deeper divergence leads to greater autocorrelation (Figure 5)— and is responsible for an additional ~40% of the constant population variation in *G*_e_. The stochasticity of the coalescent explains an additional 11% of the variation. Under a mild founder event scenario, only variation in the time of admixture (*T*_A_) has significant predictive power on the number of genomic regions, accounting for ~89% of the variation in the number of effective number of gene trees. Similarly, under an extreme founder event, *G*_e_ is strongly influenced by the time of admixture (~46%). Interestingly, in extreme founder events, variation in the coalescent process is responsible for the majority of the variation in *G*_e_ (~54%), indicating that demographic parameters (such as *T*_A_, *T*_D_ and *c*) have little predictive power for determining the number of effective number of gene trees under instances of extreme founder events.

## 3. Experimental Section

### 3.1. The Isolation and Admixture Model

The isolation and admixture (IAA) model describes the ancestral coalescence process for samples taken from a single contemporary population. Looking forward in time, the model assumes that the sampled population diverged from a second, unsampled population at time *T*_D_ in the past. Furthermore, the IAA model assumes that the two populations remain isolated until time *T*_A_, when some proportion (*c*) of ancestral lineages move from population 2 into population 1. It is convenient to think of the IAA model in terms of a traditional continent-island model of population structure, in which the island population is founded at time *T*_D_, remains isolated, and then a second colonization event from some proportion *c* of the mainland population occurs at time *T*_A_. According to this analogy, only the island population would be sampled. Figure 1a graphically illustrates the IAA model and the six associated parameters, including the effective sizes of both populations 1 and 2 (*N*_1_ and *N*_2_, respectively) and the ancestral population (*N*_A_). All times are measured in units of 4*N*_1_ generations before the present.

The IAA model can also be modified to include a founder event in one of the populations. The inclusion of a founder event, or a population bottleneck, can be traced to several motivating biological scenarios. One particularly contentious scenario corresponds to the detection of Neanderthal admixture with anatomically modern humans in Europe. In this case, we would ask whether introgression is more readily detectable if the putative admixture events happen as soon as modern humans arrive in Europe (accompanied by a reduction in effective population size) or whether introgression is more readily detectable if the admixture occurs as the modern human population is expanding and pushing Neanderthal populations to the periphery of continental Europe. A wide range of alternative biological scenarios may also be accounted for by including a founder event in the model, such as secondary contact following a period of isolation in Pleistocene glacial refugia, or repeated colonization of an island by founder mainland populations. The founder event is assumed to reduce the size of population 1 by a factor of α; we examine values of α = 0.1 and α = 0.01, corresponding to a 10-fold and 100-fold bottleneck, respectively. The reduction phase of the population bottleneck begins at time *T*_F_ and lasts until time *T*_R_, whereupon population 1 instantaneously recovers to its original effective size (Figure 1b). We explicitly consider cases for which *T*_R_ ≤ *T*_A_ ≤ *T*_F_.

To illustrate some basic properties of the IAA coalescent model, we can consider the probability that a randomly chosen gene tree harbors a deep split that may be associated with admixture between two divergent populations. For example, after *T*_A_ generations of coalescence, starting with *n* lineages, the expected number of lineages remaining (*q*) is given by the summation

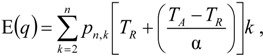
(1)
in which the weight, *p_n_*_,*k*_(*t*), is the probability of *k* lineages remaining from *n* sampled lineages after *t* generations of coalescence. This probability is
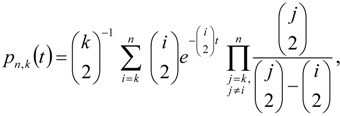
(2)
as given by Takahata and Nei [21]. In this manner, the probability of an introgression event can then be written in terms of the expected number of ancestral lineages and the admixture rate

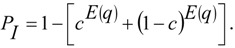
(3)

### 3.2. Choice of Model Parameters

To assess the impact of secondary contact on introgression between populations, we simulate these events across a range of biologically meaningful parameter values. We examine values of *T*_A_ between 0.01 and 0.06, in increments of 0.01 (time is measured in units of 4*N*_1_ generations). This corresponds to the recent past for typical vertebrate populations, as most instances of population admixture reported are inferred from recent events (*e.g*., post-Pleistocene secondary contact). For example, for a population with an effective population size of 10,000 and a generation time of 25 years, the simulated values of *T*_A_ correspond to 10,000 to 60,000 years before the present. Alternatively, for organisms with an effective population size of 1,000,000 and 10 generations per year, these values would range from 250 to 1500 years before the present. The time of divergence (*T*_D_) was simulated from 0.6 to 2.0, in increments of 0.2. This range was selected to encompass a wide range of likely divergence times between pairs of sister populations or species. The admixture proportion is assessed at *c* = {0.01, 0.05, 0.25, 0.50}. The values of admixture proportion were selected to account for a range of biologically interesting scenarios. Low rates of admixture (*c* = {0.01, 0.05}) are common under scenarios of hybridization along a restricted contact zone between populations and between populations that already developed strong barriers to gene exchange. Moderate admixture (*c* = 0.25) simulated widespread hybridization between populations, while high admixture (*c* = 0.50) models a hybrid swarm and may be relevant for investigators concerned with hybrid speciation.

We sample all of the above parameterizations of the IAA model assuming three distinct demographic scenarios: 1) a constant population size, 2) a mild founder event (*e.g*., a 10-fold reduction in the colonizing population), and 3) a severe founder event (100-fold reduction). The effective population sizes of the current colonizing population, the endemic population, and the ancestral population are assumed to be equal. Under the founder event scenarios, at *T*_F_ = 0.06, *N*_F_ instantaneously shrinks to a fraction of *N*_1_ (1/10 and 1/100) and at *T*_F_ = 0.01 instantaneously grows to *N*_A_ = *N*_1_. These simulations are intended to model recent, rather than ancient, population sizes changes because recent population size changes are more commonly inferred, as well as more likely to be found as a genomic signature [22].

For all simulations, we simulated a sample size of *n* = 100, a value that is large relative to the current availability of within-lineage, full chromosomal datasets, but a conservative estimate of the size sample that will be available in the near future for well studied groups, as next generation resequencing techniques become more common. We simulated our datasets with a population recombination parameter ρ = 4*N*_1_*r* (*r* is the recombination rate per site per generation) of 1000.

### 3.3. Gene Tree Summary Statistics

Coalescent simulations were performed using the computer application *ms* [23], modified to calculate and report summary statistics, the proportion of total tree time occupied by the two basal branches (*P*_BB_) and the time of the most recent common ancestor (*T*_MRCA_). Source code for the modified version of *ms* is available from the authors upon request. *P*_BB_ also corresponds to the probability that a randomly placed mutation will occur on one these two basal branches, representing a site that partitions the two basal lineages distinctively, *i.e.*, “congruent sites”, after Wall [16]. These values were determined individually for each gene tree across a simulated chromosome. To assess the size of introgressed haplotypes under our model of secondary contact, we calculated the autocorrelation of adjacent loci. This measure is indicative of the size of blocks of adjacent loci with similar values for *P*_BB_ and *T*_MRCA_. To account for the stochastic variation of the coalescent, we generated 1000 independent simulations for each unique combination of *T*_A_, *T*_D_, and *c*.

We developed scripts for the R statistical computing environment (ver. 2.10.0, RDCT 2009) (available from the authors on request) to summarize *P*_BB_ and *T*_MRCA_. In each instance, the mean and the *L* [25] autocorrelation are calculated for each summary statistic across the simulated chromosome within a replicate. These values are then summarized by taking the mean across all 1000 replicates within each parameter set. To account for the non-independence of adjacent trees, the number of effectively independent gene trees (*G*_e_) within a set of parameters was determined using the relation

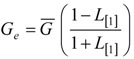
(4)
[24], where G is the mean number of trees across 1000 replicates and *L* [1] is the mean first-order autocorrelation coefficient for a particular combination of model parameters. To derive the probability density that a randomly sampled history in the genome reflects an introgression event, *φ*(*I*), the probability of an introgression event, given by equation (3), can be multiplied by the simulated values of *L* [1], averaged over all values of *T*_D_, e.g.,

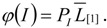
(5)

### 3.4. Multivariate analysis

We employed multivariate analyses to measure the contribution of each of the manipulated parameters to variation in summary statistics. This approach generates quantitative answers to questions such as: what portion of the variation in *T*_MRCA_ in a constant population is explained by the experimental variation in time of time of divergence (*T*_D_) of the two modeled populations? Or, what is the magnitude of the effect of varying levels of admixture (*c*) on *P*_BB_ in a population experiencing an extreme founder event?

We performed one-way ANOVA using the R statistical computing environment with the *car* package [25] to assess the impact each of the manipulated demographic parameters contributed to the variation in the calculated summary statistics. Each summary statistic (*P*_BB_ and *T*_MRCA_) was individually analyzed as the response variable to the individual and combined effects of *T*_A_, *T*_D_, and *c.* The ratio of individual sum-of-squares to total sum-of-squares was calculated to determine *r^2^*, the proportion of total variance of the summary statistic explained by the parameter or interaction between parameters. This analysis allows quantitative evaluation of the relative effects the varied parameters have on the summary statistics in each simulation. 

## 4. Conclusions

The isolation-and-admixture (IAA) model captures the dynamics of populations that come into secondary contact after some period of allopatry. If the rate of admixture in the IAA model is set to 50%, the model may also accurately predict the genomic constitution of a hybrid species. Our coalescent simulations consider several instances of the IAA model, particularly those that include the occurrence of a founder event during secondary contact. Our motivation for this study is to assess the power afforded to investigators who wish to test for past gene flow events using genome-level polymorphism data from a single species or population. One notable case where these considerations may be important is that of detecting historical human-Neanderthal gene flow. Using sequence data from a 27,000 year-old Neanderthal specimen, researchers are able to measure the number of sites for which the Neanderthal has the derived state (relative to chimpanzee) and European modern humans are polymorphic for both the ancestral and derived states [26]. These shared, derived sites should be more abundant throughout the genome if gene flow occurred, compared with the frequency expected under incomplete lineage sorting [27].

Our findings suggest that the occurrence and magnitude of a founder event in the ancestral European modern human population may produce either reduced or elevated frequencies of shared, derived sites. For example, if the founder event was strong (α = 0.01), our simulations indicate there is little chance of sampling introgressed lineages, regardless of the rate of historical admixture (Figure 3). For strong founder events, introgressed lineages are most readily detectable when the admixture event occurs very close to the time of the founder event recovery. However, the opposite is true of mild founder events. For example, a 10-fold founder event results in an increased probability of detecting admixed lineages (Figure 4) and this probability becomes still higher as the admixture time approaches the time of founder event. This dichotomy raises an interesting question about secondary contact in general. Is admixture more likely to occur early in secondary contact, when the colonizing population is small? Or, is admixture more likely to occur after the colonizing population begins to grow? As it happens, these two scenarios, in conjunction with the magnitude of founder event, result in two very different predictions about our ability to detect genetic introgression. This conclusion highlights the ongoing need to understand the basic demographic model for a given species before more complex processes, such as gene flow or natural selection, can be detected with confidence.

Ancestral lineages may be shared among populations due to either genetic introgression or incomplete lineage sorting. If polymorphism data are available from large tracts of the genome, it is possible to distinguish between these two possibilities by considering the linear arrangement of shared lineages across chromosomes. This type of inference may be increasingly plausible for a wider range of taxa in the era of next-generation sequencing technology. While shared lineages resulting from incomplete lineage sorting are expected to be randomly arranged across a genome, shared lineages due to recent gene flow or genetic introgression are expected to occur in locally autocorrelated tracts [28]. Our results indicate that the autocorrelation of shared lineages is complicated by a variety of demographic parameters. In the absence of a founder event, the rate of admixture is the primary determinant of the length of an introgressed haplotype (Table 3). Higher levels of admixture result in longer introgressed haplotypes, suggesting that this may be a diagnostic feature for testing hypotheses about hybrid speciation. In the presence of a mild founder event, the time of admixture is shown to have the greatest effect on the length of introgressed haplotypes (Figure 5).

The results of the present study are broadly applicable to a variety of biological scenarios that involve secondary contact between previously allopatric populations. Specific parameterizations of the IAA model also make predictions about the genomic distribution of gene trees expected for populations of hybrid origin. The sampling distribution of gene trees, as well as the distribution of branch lengths within trees, can be used to devise improved statistical tests for detecting reticulate evolutionary patterns. Lastly, properties such as basal branch lengths and the time to a most recent common ancestor can serve as the basis for estimating specific demographic parameters in models of reticulate evolution.
